# Potency Analysis of Medical Marijuana Products from New York State

**DOI:** 10.1089/can.2018.0037

**Published:** 2019-09-23

**Authors:** Lingyun Li, Bryan C. Duffy, Lorie A. Durocher, Mark A. Dittmar, Robert A. Acosta, Emily R. Delaney, Lei Li, Kenneth M. Aldous, David C. Spink

**Affiliations:** ^1^ New York State Department of Health, Wadsworth Center, Albany, New York.; ^2^ School of Public Health, University at Albany, State University of New York, Albany, New York.

**Keywords:** Δ^9^-tetrahydro-cannabinol, cannabidiol, cannabinoids, HPLC, medical marijuana, potency analysis

## Abstract

**Introduction:** In the United States, medical marijuana programs have been established in 29 states and the District of Columbia. In 2014, New York State (NYS) approved medical marijuana legislation, and its program became fully operational in January of 2016. Products manufactured under the auspices of the program may be used by certified patients in NYS for the treatment of 1 of 12 qualifying medical conditions. The NYS statute requires rigorous testing of each product lot manufactured in the state for its cannabinoid profile, bacterial and fungal contamination, mycotoxins, heavy metals, plant-growth regulators, and pesticides. Here, we report on the analysis of product cannabinoid profiles.

**Methods:** A method employing a simple extraction/dilution technique and reversed-phase high-performance liquid chromatography with photodiode array detection (HPLC-PDA) was developed for the analysis of 10 cannabinoids: cannabidiolic acid, cannabigerolic acid, cannabigerol, cannabidiol (CBD), tetrahydrocannabivarin, cannabinol, Δ^9^-tetrahydrocannabinol (Δ^9^-THC), cannabichromene, cannabidivarin, and Δ^9^-tetrahydrocannabinolic acid-A. The method employed internal standard quantitation and incorporated a surrogate to monitor extraction efficiency and analytical recovery.

**Results:** The HPLC-PDA method was validated using sample matrices composed of medium-chain triglycerides, hemp oil, sesame oil, and an ethanol-propylene glycol tincture. Limits of detection, limits of quantitation, accuracy, precision, and inter- and intraday reproducibility were found to be highly satisfactory. The validated method has been used to analyze over 3500 samples from over 700 lots of medical marijuana products manufactured in NYS from January 2016 through April 2018. Quality-control data showed quantitative spike recoveries and, for the analysis of samples from the same lot, the coefficients of variation for the principal analytes, Δ^9^-THC and CBD, averaged <3%. Using the HPLC-PDA method, the NYS medical marijuana products were analyzed to verify the potencies on the product labels and to determine the stability of the products.

**Conclusions:** An HPLC-PDA-based method was developed, validated, and employed to analyze 10 cannabinoids in a variety of medical marijuana products. The method has proven to be accurate, precise, stable, and very robust. Its use is an integral part of the NYS Medical Marijuana program for validation of the content and consistency of medical marijuana products.

## Introduction

The use of cannabis in the treatment of human disease dates to at least 4000 BCE.^1,2^ Due to the purported and established beneficial effects of cannabis in the treatment of numerous conditions,^3–6^ there have been increased research and development efforts into cannabinoid-based drugs. At the federal level, the Food and Drug Administration (FDA) has approved two tetrahydrocannabinol-based medications, dronabinol, the active ingredient of which is synthetic Δ^9^-tetrahydrocannabinol (Δ^9^-THC), and nabilone, a semisynthetic analog of Δ^9^-THC.^7^ These drugs are used to treat cancer patients undergoing chemotherapy who have nausea and vomiting that is not adequately controlled by conventional antiemetic treatments and for anorexia associated with weight loss in patients with AIDS. Recently, the FDA approved epidiolex, a formulation of purified cannabidiol (CBD), for the treatment of two rare and severe forms of epilepsy.^8^ In parallel developments at the state level, medical marijuana programs have been initiated in 29 states in the United States and the District of Columbia.^9^ An additional 17 states have established CBD-only programs. Since the state-operated medical marijuana and CBD-only programs are not under the purview of the FDA, it is the responsibility of the individual states to determine whether and how to monitor the potency of the products and their potential microbial and chemical contamination.

In July 2014, New York State (NYS) approved medical marijuana legislation, and its program became fully operational in January 2016. Medical marijuana is currently permitted in NYS for the treatment of conditions including amyotrophic lateral sclerosis, cancer, epilepsy, HIV/AIDS, Huntington's disease, inflammatory bowel disease, Parkinson's disease, post-traumatic stress disorder, multiple sclerosis, neuropathies, spinal cord injuries associated with spasticity, and chronic pain.^10^ The NYS program disallows the smoking of cannabis, but a variety of cannabis products including capsules, oils, tinctures, and vaporizer cartridges are available. The NYS regulations on medical marijuana require rigorous testing of each final product lot produced in the state for its cannabinoid profile, bacterial and fungal contamination, mycotoxins, heavy metals, plant-growth regulators, and pesticides. The Medical Marijuana Laboratory of the Wadsworth Center has developed, validated, and employed methods for each of these required tests. Methods using high-performance liquid chromatography with photodiode-array detection (HPLC-PDA) have proven effective for the determination of cannabinoids in cannabis plant material,^11–13^ and thus served as a basis for our procedures for the analysis of medical marijuana products.

This initial article from the Wadsworth Center Medical Marijuana Laboratory reports on the development and validation of methods for the extraction and analysis of cannabinoids in CO_2_ extract-based medical marijuana products using HPLC-PDA. We further report on the performance of our method in the analysis of over 700 lots of medical marijuana products during the first 2 years of the NYS Medical Marijuana Program and on stability studies of opened and unopened products.

## Materials and Methods

### Chemicals and standards

Certified cannabinoid reference standards (at 1 mg/mL in methanol) including cannabidiolic acid (CBDA), cannabigerolic acid (CBGA), cannabigerol (CBG), CBD, tetrahydrocannabivarin, cannabinol (CBN), Δ^9^-THC, cannabichromene (CBC), cannabidivarin (CBDV), and Δ^9^-tetrahydrocannabinolic acid-A (THCA) were purchased from Cerilliant (Round Rock, TX). Second-source standards of CBN, CBD, and Δ^9^-THC were from Restek (Bellefonte, PA). CBD hemp oil was purchased from HempMeds (Poway, CA). Norgestrel, 4-pentylphenyl 4-methylbenzoate (PPMB), ammonium formate, formic acid, and sesame oil were from Sigma-Aldrich (St. Louis, MO). Medium-chain triglyceride (MCT) oil was from Warner Graham (Cockeysville, MD). Propylene glycol was from J.T. Baker (Central Valley, PA). Ammonium formate, formic acid, methanol, acetonitrile, and water were HPLC grade. All other reagents used were analytical grade. Bulk cannabis plant material was obtained from the National Institute on Drug Abuse (NIDA), National Institutes of Health, Bethesda, MD.

### Supercritical fluid extraction of cannabinoids

In-house extractions were performed with 10–15 g of ground cannabis plant material from NIDA. Weighed samples were placed in 25-mL stainless steel extraction vessels, and a Waters MV-10 ASFE supercritical extractor system controlled by ChromScope^®^ software was used for supercritical CO_2_ (>99.99% purity) extraction without the addition of modifiers. The CO_2_ flow rate was 2.0 mL/min at 57°C with a controlled pressure of 25 MPa. Absolute ethanol at a flow rate of 4.0 mL/min was used to elute the extracted materials. The optimal extraction time was determined to be 3 h. Extracted cannabinoids were concentrated using a rotary evaporator mounted in a 57°C water bath. After ethanol removal, the concentrated cannabis extract was either analyzed directly or incubated at 110°C for varying times to evaluate the level of decarboxylation of the cannabinoid acids. These extracts were used in HPLC-PDA method development.

### Sample preparation for HPLC analysis

A dilution/extraction method was used for all samples. Sample matrix or medical marijuana product (10–200 mg; the sample amount was dependent on the cannabinoid concentrations of the various products) was weighed to ±0.01 mg in a tared centrifuge tube. A 10-μL aliquot of a 50 mg/mL PPMB surrogate solution was spiked into each sample. Methanol (20.0 mL) was added, and the solution was mixed on a laboratory shaker for 30 min to extract the cannabinoids. Aliquots (1 mL) of the extracts were transferred to 1.5-mL centrifuge tubes and, if cloudiness or precipitate was observed, the tubes were centrifuged at 12,000 *g* for 5 min. Depending on the expected cannabinoid concentrations, supernatants were diluted up to 100-fold with methanol to ensure that the cannabinoid concentrations determined fell within the ranges of the calibration curves. A 500-μL aliquot of the norgestrel internal standard at 10 μg/mL was added to 500 μL of diluted sample for analysis.

### Cannabinoid analysis using HPLC-PDA

Two identical HPLC systems (Shimadzu, Kyoto, Japan), each consisting of a model SIL-20ACxR autosampler, a model FCV-11A2 solvent selector, model LC-20ADxR pumps, a DGU-20A3 micro vacuum degasser, a model column oven, and a model SPD-M20A PDA detector were used. Data acquisition and processing were controlled by Lab Solutions^®^ software (version 5.73). The autosamplers were maintained at 4°C; injection volumes were 10 μL. HPLC separations were performed on Poroshell^®^ 120 columns, 3.0×150 mm with 2.7 μm particle size (Agilent, Santa Clara, CA). During analysis, the columns were maintained at 30°C. Elution of the cannabinoids was monitored at 227 nm. Mobile phase A consisted of 0.1% (v/v) formic acid in 25 mM aqueous ammonium formate; mobile phase B was 0.1% (v/v) formic acid in acetonitrile. The HPLC flow rate was 0.625 mL/min. The elution program was as follows: from 0 to 18 min, mobile phase B was at 73% for isocratic elution; mobile phase B was increased to 100% at 19 min and was held at 100% until 21 min for column wash; mobile phase B was then returned to 73% at 21.1 min. The column was re-equilibrated at 73% B for 2.9 min, resulting in a total run time of 25.0 min. An analytical batch consisted of a maximum of 20 samples. Quality control (QC) samples analyzed with each batch of samples were a method blank, a method blank spike, and a matrix spike.

### Method validation

The method was validated using procedures approved by the NYS Environmental Laboratory Approval Program (ELAP), which subsequently certified the method.^14^ The limit of detection (LOD) for each analyte was determined at the 99% confidence level^15^ from the analysis of 7 blank samples that were fortified with low levels of 10 cannabinoids and the surrogate. The limit of quantitation (LOQ) for each cannabinoid was defined as five times the LOD, provided that this value was not below the lowest concentration calibrant of the standard curve (0.19 μg/mL), in which case the lowest concentration calibrant was assigned as the LOQ concentration.

Calibration curves were prepared with cannabinoid standards and PPMB at concentrations of 0.19, 0.56, 1.67, 5.0, 15.0, and 45.0 μg/mL. Internal standard quantitation was used, whereby the ratios of peak areas of the analytes to those of the internal standard served as the quantitative measure. For determination of method accuracy, QC samples spiked with low (1.67 μg/mL), mid (5.0 μg/mL), and high levels (45 μg/mL) of cannabinoids were analyzed. Initial stability studies were performed using repeated analysis of samples maintained for 70 h at ambient temperature or 1 week at 4°C. Matrices used for method evaluation were MCT oil, olive oil, and a tincture consisting of a 1:1 (v/v) mixture of ethanol and propylene glycol. Initial matrix validations were performed by spiking with hemp oil, which contained CBD as its main cannabinoid, but also contained low levels of other cannabinoids.

### Analysis of medical marijuana products

Since the inception of the NYS Medical Marijuana program, a variety of products have been developed and submitted to our laboratory for testing. The Registered Organizations (ROs), the approved medical marijuana producers in NYS, kindly provided potential excipients and placebo/matrix material for analysis to ascertain potential interferences and to identify problematic components affecting cannabinoid recovery. Once products were approved by the program and were in production, each final lot required testing before distribution to patients. The Medical Marijuana Laboratory received 5–14 samples per lot under chain of custody. Each sample received was analyzed for potency. Sample types included capsules, tablets, sublingual oral solutions, tinctures, and vaporizer cartridges. To determine the stability of products at 30 and 60 days after opening, portions of the original samples were analyzed after storage under conditions described on the labels. For unopened product stability determinations at 120, 240, and 360 days postpackaging, additional samples from each lot were obtained from the ROs. All medical marijuana samples were secured in a safe within a card-access laboratory before and after analysis. Complete controlled substance inventories were maintained, and excess material after testing was destroyed using a method approved by the NYS Department of Environmental Conservation.

## Results and Discussion

**FIG. 1.**
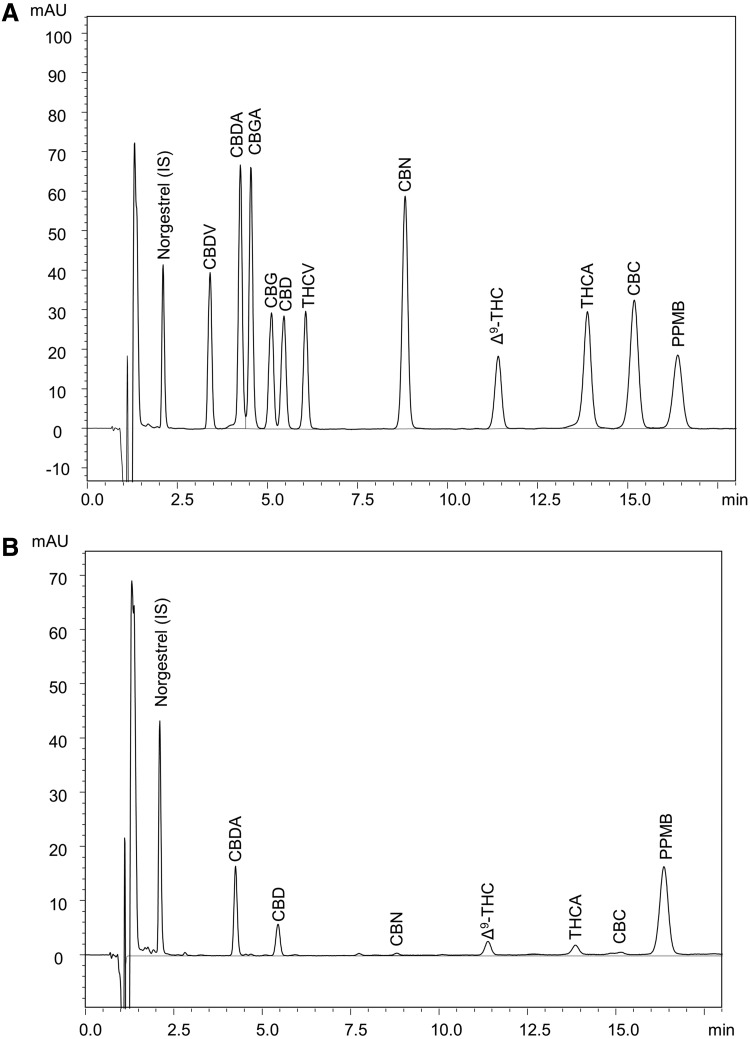
Analysis of cannabinoids using HPLC-PDA. **(A)** Chromatogram of calibration standards, internal standard, and surrogate, each at 15 μg/mL, with detection at 227 nm. **(B)** Representative chromatogram from the analysis of a supercritical CO_2_ extract of National Institute on Drug Abuse bulk cannabis material before decarboxylation. HPLC-PDA, high-performance liquid chromatography with photodiode array detection; CBDA, cannabidiolic acid; CBGA, cannabigerolic acid; CBG, cannabigerol; CBD, cannabidiol; THCV, tetrahydrocannabivarin; CBN, cannabinol; Δ^9^-THC, Δ^9^-tetrahydrocannabinol; THCA, Δ^9^-tetrahydrocannabinolic acid-A; CBC, cannabichromene; CBDV, cannabidivarin; PPMB, 4-pentylphenyl 4-methylbenzoate.

Analysis of the 10 major cannabinoids in cannabis together with the internal standard, norgestrel, and the surrogate, PPMB, with detection at 227 nm is shown in Figure 1. Complete resolution of the 10 cannabinoids in the calibration standard was obtained except for the CBDA/CBGA pair, which was 95% resolved (Fig. 1A). This resolution was adequate for the quantitation of the two cannabinoids. Norgestrel and PPMB were well resolved from the cannabinoids. The analysis of a supercritical fluid CO_2_ extract of the NIDA cannabis material is shown in Figure 1B. The sample had not been subjected to decarboxylation, and therefore showed significant amounts of CBDA and THCA. Importantly, no interferences were observed that would confound the analysis of the cannabinoids, surrogate, or internal standard.

Calibration curves for the 10 cannabinoids and PPMB showed excellent linearity, with coefficients of determination (*R*^2^) for each analyte exceeding 0.9995 (Table 1). Analytical precision was determined by analysis of 8 replicates of samples spiked with the 10 cannabinoids and PPMB, each at 1.8 μg/mL. Coefficients of variation (%CV) from these analyses ranged from 0.87% to 5.27%. LODs and LOQs for the cannabinoids (Table 1) indicated that the sensitivity of the method was adequate for the determination of cannabinoids in medical marijuana products. Norgestrel and PPMB were initially investigated for use as the internal standard and surrogate respectively based on favorable chromatographic properties and molar absorptivity at 227 nm. Norgestrel and PPMB were found to be quite stable during analytical procedures and under normal storage conditions. Storage of the stock solutions of norgestrel and PPMB for 70 h at ambient temperature or 1 month at 4°C resulted in <1% change in their concentrations. The efficacy of norgestrel as an internal standard is evidenced by the linearity of the calibration curves, which are based on analyte/internal standard area ratios, and by subsequent accuracy, precision, reproducibility, and stability studies of the method. PPMB showed comparable values for calibration curve *R*^2^, LOD, and LOQ to those of the cannabinoids (Table 1). Further studies of the accuracy, precision, reproducibility, and stability of the method verified the suitability of PPMB as the surrogate.

**Table 1.** Calibration Curve Linearity and Values of Limit of Detection and Limit of Quantitation for Ten Cannabinoids and the 4-Pentylphenyl 4-Methylbenzoate Surrogate

Analyte	*R*^2^	LOD (μg/mL)	LOQ (μg/mL)
CBDV	0.9997	0.063	0.32
CBDA	0.9999	0.025	0.19
CBGA	0.9996	0.041	0.20
CBG	0.9998	0.051	0.25
CBD	0.9997	0.059	0.30
THCV	0.9998	0.027	0.19
CBN	0.9996	0.035	0.19
Δ^9^-THC	0.9999	0.101	0.50
THCA	0.9998	0.158	0.79
CBC	0.9998	0.037	0.19
PPMB	0.9997	0.046	0.23

Calibration cures prepared with 0.19, 0.56, 1.67, 5.0, 15.0, and 45.0 μg/mL of each cannabinoid and PPMB were subjected to linear regression analysis, providing the values of *R*^2^ as shown. Values for LOD and LOQ were determined as described in section “Materials and Methods”.

LOD, limit of detection; LOQ, limit of quantitation; CBDA, cannabidiolic acid; CBGA, cannabigerolic acid; CBG, cannabigerol; CBD, cannabidiol; THCV, tetrahydrocannabivarin; CBN, cannabinol; Δ^9^-THC, Δ^9^-tetrahydrocannabinol; THCA, Δ^9^-tetrahydrocannabinolic acid-A; CBC, cannabichromene; CBDV, cannabidivarin; PPMB, 4-pentylphenyl 4-methylbenzoate.

The HPLC-PDA method was validated using sample matrices that were comparable to those that were to be encountered in the NYS Medical Marijuana Program. Initial matrix validation studies involved spiking hemp oil into sesame oil and the ethanol-propylene glycol tincture and comparing the analytical recoveries with those of the original hemp oil. The hemp oil contained 19.1% (wt/wt) CBD, but also contained measurable amounts of CBDV, CBDA, CBG, Δ^9^-THC, and CBC. Analytical recoveries were ≥98.2% for each of the six cannabinoids derived from the hemp oil spiked into sesame oil and the tincture solution (data not shown). Commercial MCT oil was also investigated as a matrix, since this material is used as an excipient for some products formulated into gel capsules and other products. Results from the analysis of low- (1.67 μg/mL), mid- (5.0 μg/mL), and high-level (45 μg/mL) spikes of cannabinoids and PPMB into MCT oil are shown in Table 2. At each of the three levels, measured cannabinoid concentrations were all within ±10% of the spike levels, indicating no significant matrix effect.

**Table 2.** Cannabinoid and Surrogate Recovery with Medium-Chain Triglyceride Oil as the Matrix

Spike concentration	1.67 μg/mL	5.00 μg/mL	45.0 μg/mL
**Analyte**	**Recovery (%)**	**Recovery (%)**	**Recovery (%)**
CBDV	107.6±1.1	104.4±0.3	98.4±0.2
CBDA	109.5±1.8	106.3±0.6	96.3±0.1
CBGA	109.7±2.0	109.7±0.5	99.1±0.3
CBG	106.4±0.9	104.2±0.3	98.78±0.04
CBD	106.8±1.5	103.6±0.6	99.03±0.05
THCV	104.3±2.4	103.1±0.5	99.1±0.2
CBN	106.0±0.9	103.1±0.4	99.3±0.3
Δ^9^-THC	109.4±1.8	104.5±0.9	98.7±0.6
THCA	97.7±5.3	101.9±3.1	100.2±1.4
CBC	107.3±1.5	104.3±0.4	99.0±0.2
PPMB	107.6±2.6	102.9±1.0	99.3±0.2

The 10 cannabinoids and PPMB surrogate were spiked into MCT oil at the levels indicated, and the samples were analyzed using HPLC-PDA. Data shown are from five replicates±relative standard deviation expressed as a percentage of the spike level.

HPLC-PDA, high-performance liquid chromatography with photodiode array detection; MCT, medium-chain triglyceride.

**FIG. 2.**
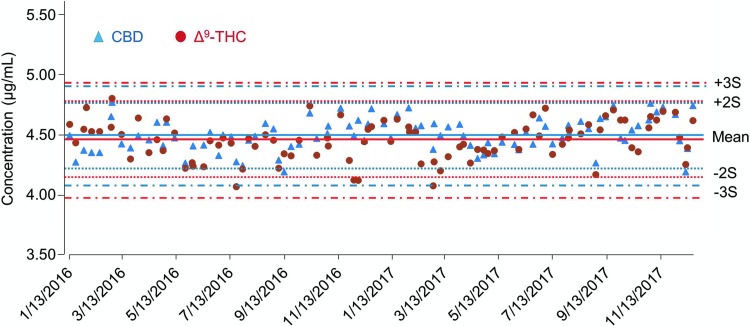
QC monitoring of the analysis of Δ^9^-THC and CBD over a 2-year period. Shown are Levey-Jennings plots of the analytical values determined for Δ^9^-THC and CBD in the QC samples, spiked at 4.5 μg/mL, analyzed from January 2016 through December 2017. QC, quality control; S, standard deviation.

Following method validation and certification by ELAP,^14^ the method was used to analyze medical marijuana products from the NYS ROs. From January 2016 through April 2018, over 1300 medical marijuana product samples including tinctures, oils, capsules, and vaporizer cartridge oils were assayed. While potencies on product labels referred only to, on a mass basis, total Δ^9^-THC (Δ^9^-THC + THCA) and total CBD (CBD + CBDA), complete profiles of the 10 cannabinoids shown in Figure 1 were determined. Over the 2-year period reported here, the accuracy and precision of the method remained unchanged as determined by QC data from each analytical batch. Figure 2 shows the compilation of QC data for the analysis of Δ^9^-THC and CBD. The spike recoveries were determined to be within ±10% of the theoretical value for both THC and CBD. There was no trend in recovery during the analysis period. Similar results were obtained for the other cannabinoids (data not shown).

**FIG. 3.**
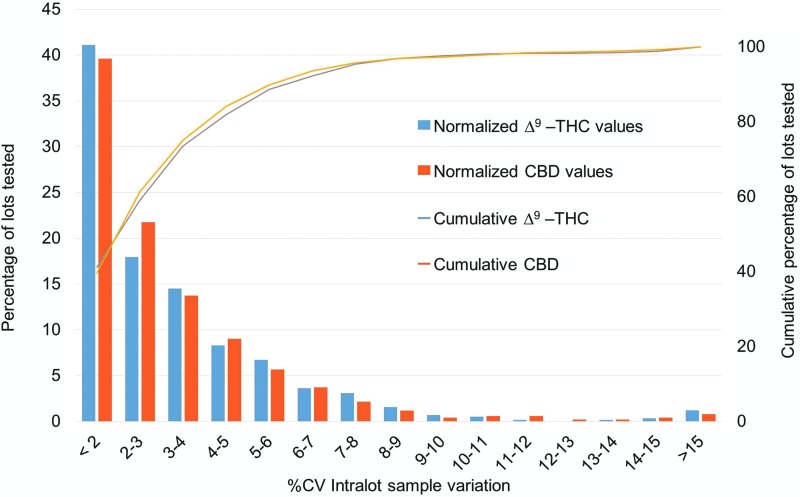
Distribution of the intralot %CV values from the analysis of total Δ^9^-THC and total CBD concentrations in all medical marijuana product lots analyzed in 2016 and 2017 with *n*≥5 samples per lot. %CV, coefficient of variation.

The high precision of the method was also maintained over the 2-year period. The %CV for the total Δ^9^-THC and total CBD normalized by the percentage of all lots tested during 2016–2017 shows remarkable intralot reproducibility (Fig. 3). The average percentage of each cannabinoid was determined from 5–14 samples per final lot and multiplied by the gross dose weight to report as weight percentages. When the cannabinoid percentage was less than the LOQ, the value of “0” was used in the total cannabinoid calculation. The %CV of each lot was calculated and binned by increasing percent variation. Most of the product lots had a %CV of <3%. The percentage of lots progressively decreased with increasing %CV, reflecting the normal statistical distribution of the combined contributions of method error and product inhomogeneity. The cumulative total Δ^9^-THC and total CBD values increase similarly, indicating no method or product reproducibility bias by the major cannabinoid. Overall, the analytical method performs well for both total Δ^9^-THC and total CBD potency determinations across many product types and concurrently demonstrates the homogeneity of the product samples for various medical marijuana products available in NYS.

**FIG. 4.**
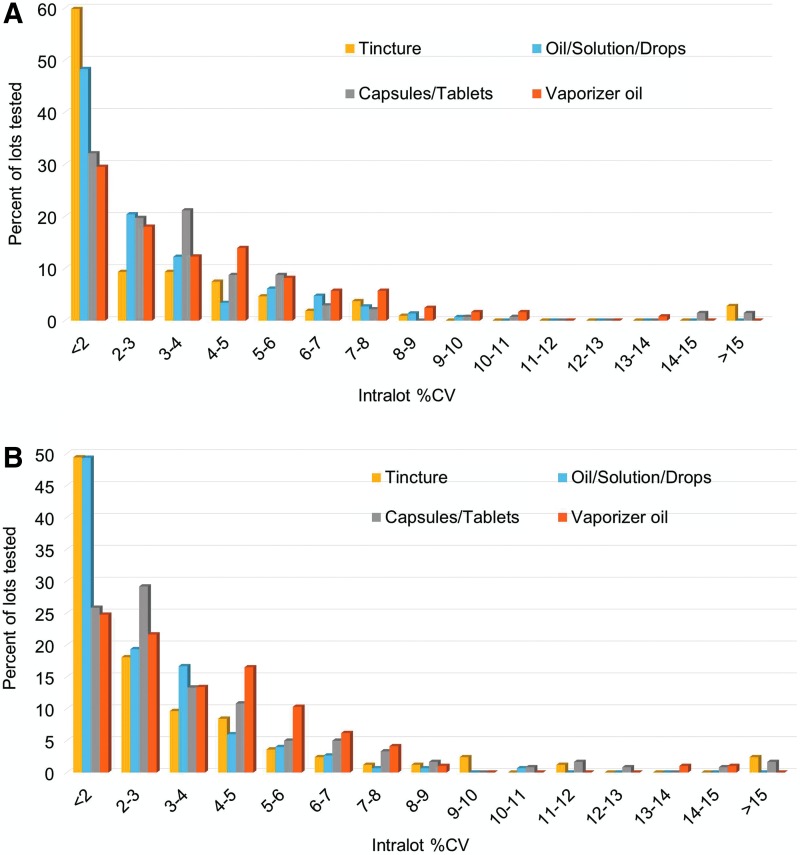
Distribution of the intralot %CV values from the analysis of total Δ^9^-THC and total CBD concentrations in all medical marijuana product lots analyzed in 2016 and 2017 segregated by product type. **(A)** The distribution of %CV for the analysis of Δ^9^-THC in the product types with *n*≥5 samples per lot. **(B)** The distribution of %CV for the analysis of CBD in the product types with *n*≥5 samples per lot.

A detailed breakdown of analytical precision by product type (Fig. 4) shows similar reproducibility, but with some matrix-specific exceptions. Although many product types are available in NYS, they can be separated into four major categories: tinctures, oils/solutions/drops, capsules/tablets, and vaporizer oils. Final product potency data from all ROs were sorted into these four categories. The product type was observed to influence the reproducibility in terms of %CV. The method reproducibility is predicted to be constant, while individual product homogeneity likely has greater variability across the product types.

The ethanol-based tinctures were found to have the greatest intralot reproducibility, followed closely by the oil/solution/drops products. The slight difference between the total Δ^9^-THC and total CBD in the <2 and 2–3%CV bins may be due to the varying numbers and types of tinctures. The capsule/tablet products, which contained both oil-based and powder-based formulations, had distinctly larger and broader %CV distribution than the oral liquid-based products, but certainly not what would be considered high variation. The vaporizer oils showed the highest variation. Several sources of both method error and inhomogeneity could contribute to this observation. Oil samples are removed from the vaporizer pens/cartridges using a syringe or spatula. The vaporizer oils are the most concentrated, require the smallest dose, and are highly viscous; therefore, increased weighing and dilution errors occur in their analysis using this and similar potency methods. Inhomogeneity due to vaporizer cartridge packaging is also possible. The viscous vaporizer oils are in contact with surfaces of the cartridge materials, including metals, glass, plastics, wicks, and seals. Additionally, liquid exposed to air inside the cartridge is subject to oxidation and evaporation of the more volatile fractions of natural terpenes and excipients. This combination of factors, and the large variation in product formulations and vaporizer devices among the ROs, may lead to larger variations in potency measurements than for other forms.

An additional requirement of the NYS Medical Marijuana Program is the determination of stabilities of total Δ^9^-THC and total CBD in the products. Overall, product stability was found to be excellent. The mean stabilities for each product type at 30 and 60 days after opening were ≥98.8%. Unopened product stabilities at 120, 240, and 360 days postpackaging exceeded 83% for total Δ^9^-THC and total CBD in all product types (Table 3).

**Table 3.** Stability of Total Δ^9^-Tetrahydrocannabinol and Total Cannabidiol in Medical Marijuana Products

Product type	Cannabinoid	Day 30	Day 60	Day 120	Day 240	Day 360
Tincture	Total Δ^9^-THC	99.5±3.9	99.0±3.0	98.4±5.5	93.6±7.8	96.4±8.4
(76 Lots)	Total CBD	99.7±3.7	99.8±3.1	100.1±5.8	99.3±6.0	98.9±8.7
Oil/oral solution	Total Δ^9^-THC	100.7±3.4	97.4±5.2	94.0±8.5	87.5±17.2	90.6±10.8
(53 Lots)	Total CBD	101.3±3.7	99.0±4.6	99.1±8.0	101.1±18.3	95.2±13.7
Capsule/tablet	Total Δ^9^-THC	97.1±3.4	99.2±2.5	95.2±4.8	95.3±8.9	101.4±12.4
(89 Lots)	Total CBD	99.7±1.6	101.2±1.1	99.9±4.1	95.3±2.4	102.1±3.8
Vaporizer oil	Total Δ^9^-THC	99.9±4.8	98.8±5.5	96.3±8.0	84.7±9.4	83.4±9.0
(111 Lots)	Total CBD	100.6±2.4	100.1±4.5	100.0±5.8	95.5±6.6	98.9±9.8

Stabilities of total Δ^9^-THC and total CBD at the indicated times, expressed as the percentage of their initial values (±relative standard deviation), were determined for each lot tested and segregated according to product type. The 30- and 60-day stability determinations were on opened products, and the 120-, 240-, and 360-day stability determinations were on unopened products.

## Conclusions

An HPLC-PDA-based analytical method was developed, validated, and used to analyze 10 cannabinoids in a variety of medical marijuana products. The method has proven to be accurate, precise, stable, and very robust. The validated method was used to analyze over 3500 samples from over 700 lots of medical marijuana products produced in NYS from January 2016 through April 2018 for confirmation of label potencies and product stabilities. Statistical evaluations of QC data and the analysis of multiple samples from the same medical marijuana production lot verified the accuracy and precision of the method on a continuing basis. The HPLC-PDA method reported here has been and continues to be an integral part of the NYS Medical Marijuana Program.

## Abbreviations Used

Δ^9^-THC: Δ^9^-tetrahydrocannabinol
%CV: coefficient of variation
CBC: cannabichromene
CBD: cannabidiol
CBDA: cannabidiolic acid
CBDV: cannabidivarin
CBG: cannabigerol
CBGA: cannabigerolic acid
CBN: cannabinol
ELAP: Environmental Laboratory Approval Program
FDA: Food and Drug Administration
HPLC-PDA: high-performance liquid chromatography with photodiode array detection
LOD: limit of detection
LOQ: limit of quantitation
MCT: medium-chain triglyceride
NIDA: National Institute on Drug Abuse
NYS: New York State
PPMB: 4-pentylphenyl 4-methylbenzoate
QC: quality control
*R*^2^: coefficient of determination
RO: Registered Organization
THCA: Δ^9^-tetrahydrocannabinolic acid-A
THCV: tetrahydrocannabivarin
